# Pharmacokinetic Study and Optimal Formulation of New Anti-Parkinson Natural Compound Schisantherin A

**DOI:** 10.1155/2015/951361

**Published:** 2015-05-17

**Authors:** Fei Sa, Bao Jian Guo, Sai Li, Zai Jun Zhang, Hok Man Chan, Ying Zheng, Simon Ming Yuen Lee

**Affiliations:** ^1^State Key Laboratory of Quality Research of Chinese Medicine and Institute of Chinese Medical Sciences, University of Macau, Taipa, Macau; ^2^Institute of New Drug Research, Guangdong Province Key Laboratory of Pharmacodynamic Constituents of Traditional Chinese Medicine & New Drug Research, College of Pharmacy, Jinan University, Guangdong, China

## Abstract

Our recent studies showed that schisantherin A (StA) is a promising candidate for PD treatment, but the pharmacokinetic profile of StA is largely unknown. The effects of different formulations on the pharmacokinetics and bioavailability of StA were investigated by HPLC equipped with a vacuum degasser, a quaternary pump, a manual sampler, and an ultraviolet detector. The absolute bioavailability of StA in nanoemulsion formulation was significantly increased from 4.3% to 47.3%. To the best of our knowledge, this is the first report of absolute bioavailability for StA in rats and successful increase of bioavailability of StA by nanoemulsion formulation. The pharmacokinetic profiles of StA could be significantly improved by a safe nanoemulsion formulation. This study provides a successful example of advanced delivery system for improving the bioavailability of potential central nervous system (CNS) drug candidate with poor solubility. This novel approach could be an effective alternative solution to overcome the shortcomings of conventional poor drug delivery of CNS drugs. The results of present study not only indicate that StA has potential to be developed as a promising oral therapeutic agent for the management of PD but also shed light on novel way to improve bioavailability of PD drugs.

## 1. Introduction

Parkinson's disease (PD) is a slow-progressing neurodegenerative disease, affecting 4–6 million people worldwide, which is primarily caused by loss of dopaminergic (DA) neurons in the substantia nigra pars compacta (SNpc). PD is characterized by debilitating symptoms such as resting tremor, rigidity, and bradykinesia [1]. Currently, levodopa remains the most effective treatment for PD symptoms. Although dopamine replacement may alleviate disease symptomatology, under mild and medium pathological conditions, a substantial number of treated patients exhibit reduced response efficacy and even increased adverse effects. Thus, from a therapeutic point of view, an effective agent with disease-modifying is urgently needed. Chinese medicinal herbs possess a large and natural chemical diversity. They are an important potential source of efficient, alternative ways of developing neurodegenerative disease-modifying drugs, compared with conventional high-throughput screening in a compound library. Flavonoids, indoles, and tetramethylpyrazine are some examples of successful, novel neuroprotective candidates identified in botanical materials and selected based on ethnomedical knowledge [2–5].

The scaffold structure of the dibenzocyclooctadiene lignans exhibited protective effects against neuronal cell death in stroke and other neurodegenerative diseases and cognitive impairments in Alzheimer's disease. Schisantherin A (StA), a dibenzocyclooctadiene lignan from the fruit of* Schisandra chinensis *(Turcz.) Baill, has been used as an antitussive, tonic, and sedative agent under the name of Wuweizi in Chinese traditional medicine. StA is believed to have a wide variety of therapeutic effects including antihepatotoxic effects, anticarcinogenic effects, and antioxidant effects [6–8]. In our recent completed studies, we systematically investigated the capacity of five dibenzocyclooctadiene lignans (namely, schisandrin A, schisandrin B, schisandrin C, schizandrol A, and schisantherin A) to protect neuroblastoma SH-SY5Y cells from toxicity induced by 6-OHDA or MPP^+^ [9, 10]. Among the tested dibenzocyclooctadiene lignans, only StA significantly protected SH-SY5Y cells against 6-OHDA- or MPP^+^-induced cell death. The potential structure-activity relationship revealed a large benzoyloxy substituent group, on the cyclooctadiene ring in StA, and is probably functionally important for its neuroprotective activity. Moreover, StA conferred significant protection against 6-OHDA- or MPTP-induced damage in TH-positive dopaminergic neurons in PD zebrafish and mice model. The in-depth mechanistic assessment demonstrated that StA exhibited neuroprotection against MPP^+^ through the regulation of two distinct pathways including CREB-mediated Bcl-2 expression and PI3K/Akt survival signaling. All our* in vitro* and* in vivo* data provide the strong evidence of StA's promising anti-PD activity. Therefore, StA has been suggested for use as potential therapeutic candidate for treating neurodegenerative disease.

Those promising results prompted us to investigate the pharmacokinetics of StA and also further develop new oral formulations to improve the bioavailability of this new anti-Parkinson natural compound StA for the treatment of PD.

## 2. Materials and Methods

### 2.1. Chemicals and Reagents

Schisantherin A (batch number E-0134) was purchased from Shanghai Tauto Biotech Co., Ltd. (Shanghai, China). The purity of all the compounds used in this study was >98%. Soybean oil was purchased from Sigma (St. Louis, USA). Solutol HS 15 was purchased from BASF (Ludwigshafen, Germany). Acetonitrile and methanol (HPLC grade) were purchased from Hanbang Company (Jiangsu, China). All other reagents were obtained from Sigma-Aldrich (St. Louis, USA) unless stated otherwise. Ultrapure water was prepared by a Milli-Q Plus water purification system (Millipore, Bedford, MA, USA).

### 2.2. Instrumentation and Chromatographic Conditions

Quantitative analysis of StA was completed on an Agilent series 1260 HPLC apparatus (Agilent Technologies, Santa Clara, CA, USA) equipped with a vacuum degasser, a quaternary pump, a manual sampler, and an ultraviolet detector. RP Phenomenex C-18 column (250 mm × 4.6 mm, 5 *μ*m) was used at 35°C with a mobile phase of water and methanol (83 : 17, v/v). The flow rate was 1 mL/min. The monitoring wavelength of StA was 230 nm.

### 2.3. Method Validation

The method was validated for selectivity, accuracy, precision, and recovery. The selectivity of the method was evaluated by comparing the chromatograms of blank plasma samples from experimental rats with that of blank plasma spiked with standard solutions and rat plasma samples after StA administrated. The accuracy and the precision were expressed as (mean observed concentration/spiked concentration) × % and relative standard deviation (RSD%), respectively. Extraction recovery of StA in plasma sample was calculated by comparing the peak areas of the extracted QC samples to that of the unextracted standard solutions at equivalent concentrations. The calibration curve was plotted using a 1/*x* ^2^ weighted linear regression of the peak area of StA versus its plasma concentration.

### 2.4. Pharmacokinetic Study of StA in Rat

All animal experiments were conducted according to the ethical guidelines for animal experiments of Jinan University. The experimental protocols were approved by the Ethics Committee for Animal Experiments of Jinan University (permit number 20110810). Male Sprague-Dawley rats (SD rats) with body weight between 220 and 250 g and age between 6 and 7 weeks were supplied by Guangdong Medical Experiment Animal Center (Guangzhou, China). On the day before the experiment, a polyethylene catheter (0.50 mm ID, 1.00 mm OD) was cannulated into the right jugular vein under 10% chloralic hydras (i.p. 250 mg/kg) anesthesia. After surgery, the rats were placed individually in cages and allowed to recover for at least 12 h.

In order to calculate and improve the oral bioavailability of StA, StA has been formulated into different formulations including nanoemulsion for comparison. Rats were randomly divided into 3 groups (three rats per group) given dosing and formulation regimens as follows.

*Formulation 1 (tPAP formulation)*. StA was dissolved in a mixture of Tween-80, propanediol, anhydrous ethanol, and polyvinyl acetate (0.8 : 0.8 : 0.8 : 8.2, v/v). And then, the suspension of StA was orally administrated to rats at 300 mg/kg.

*Formulation 2 (dPS formulation)*. StA was absolutely dissolved in mixture of DMSO, PEG400, and saline (1 : 1 : 1). Before an i.v. bolus injection of StA at 30 mg/kg, the micropore filter membrane (0.22 *μ*m) was used to remove the impurities in this solution.

*Formulation 3 (nanoemulsion formulation)*. StA was prepared as nanoemulsion for i.v. and i.g. at 30 mg/kg. The concentration of StA nanoemulsion was 4.9 mg/mL. Briefly, crystalline StA powders were completely dissolved in soybean oil containing Solutol HS 15 (52.6%, w/w) as a nonionic surfactant and by stirring the materials at ambient temperature to obtain 1% (w/w) StA oil phase. Oil-in-water nanoemulsion was prepared by mixing above oil phase (32.2%, w/w) with aqueous phase (67.8%, w/w) at ambient temperature using a sonicator with a 3 mm probe (LCD Ultrasonic Cell Crusher, Bilon Instruments, Shanghai, China) with an output power of 75 W. The freshly prepared macroemulsion was then sealed in aluminum foil covered 50 mL test tubes and stored at ambient temperature. The mean particle diameter of nanoemulsion was measured using dynamic light scattering (Nano-ZS, Malvern Instruments, Worcestershire, UK). The nanoemulsion was diluted 10 times with aqueous solution prior to analysis. The nanoemulsion had a relative small mean particle size of 300.7 ± 2.7 nm (*n* = 3) and the PDI values were less than 0.34.

After administration of StA, blood samples were collected at 0.033, 0.167, 0.5, 1, 2, 4, 8, 12, 24, and 36 h, respectively. Plasma samples were obtained after centrifugation of the collected blood samples at 3,000 rpm for 5 min. Subsequently, in 100 *μ*L plasma which was taken from the supernatant, 200 *μ*L of 50% acetonitrile and 50% methanol were added. After vortexing for 20 s and centrifuging at 12,000 rpm for 10 min, the supernatant was collected and filtered through a 0.22 *μ*m membrane, of which 20 *μ*L was injected into the chromatographic system for analysis. Pharmacokinetic parameters were calculated with DAS 3.0 software (Shanghai, China).

## 3. Results and Discussion

### 3.1. Method Validation

The selectivity of the method was demonstrated by typical chromatograms of blank plasma, blank plasma spiked with standard solution at the concentration of 1 *μ*g/mL, and plasma sample obtained after administration of StA. There were no significant endogenous peaks directly interfering with the detection of analytes in plasma, respectively (data not shown). The retention time of StA was about 6.1 min. Under the established analysis method, the lower limit of detection (LOD) and lower limit of quantitation (LOQ) of StA were 0.02 and 0.025 *μ*g/mL for plasma sample. In the range of 0.25–25 *μ*g/mL for plasma samples, the calibration curve of StA showed good linearity (*r* ^2^ ≥ 0.999) using a 1/*x* ^2^ weighted linear regression. The intraday and interday precision and accuracy in rat plasma were evaluated by using the low, medium, and high concentration of QC samples (Table 1). The accuracy values were from 101.9 ± 2.32% to 104.5 ± 4.41% in the plasma. The overall plasma extraction recovery of StA at both medium and high concentration (*n* = 6) surpassed 88.3 ± 4.53% (Table 1).

### 3.2. Pharmacokinetic Study

**Figure 1:**
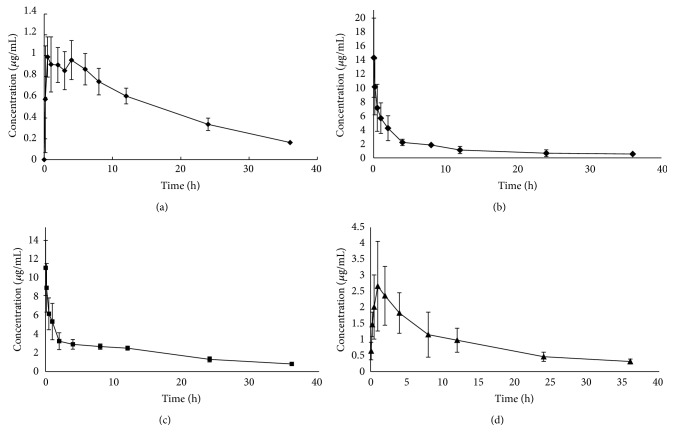
Mean plasma concentration-time profile of StA in three different formualtions by intravenous or intragastric administration (Mean ± SD, *n* = 3). (a) Intragastric administration of TPAP formulation; (b) intravenous administration of DPS formulation; ((c) and (d)) intragastric and intravenous administration of nanoemulsion formulation.

The concentration-time profiles of StA in rat plasma were presented in Figure 1. Noncompartmental model of DAS 2.0 program was used to determine the major pharmacokinetic (PK) parameters. It showed that StA's PK profile exhibited two processes of rapid distribution and slow excretion after administration (Figure 1). The *t*_1/2_ of all tested dosage forms in present study was ranged from 12 to 16 h. As shown in Table 2, when the doses of StA of different formulations were normalized, the value of AUC_(0-*t*)_/dose of StA of TPAP formulation was significantly lower than that of nanoemulsion formulation after intragastric administration. It suggests that the TPAP formulation of StA was not efficient for oral absorption. In order to increase the bioavailability of StA, we had developed nanoemulsion of StA (nanoemulsion formulation). Compared with TPAP formulation, the absolute bioavailability of StA in nanoemulsion formulation significantly increased from 4.3% to 47.3%. To the best of our knowledge, this is the first report of absolute bioavailability for StA in rats and successful increase of bioavailability of StA by nanoemulsion formulation.

## 4. Discussion

**Figure 2:**
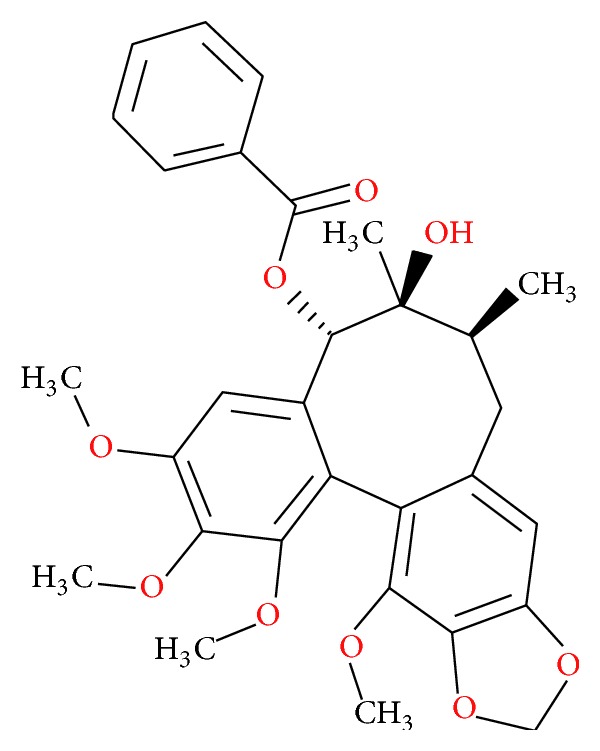
Chemical structure of the schisantherin A.

After decades of use, levodopa remains the mainstay of PD treatment. However, because PD by its nature is a progressive disease and because there are no medications proven to delay advancement of symptoms, patients are subject to a therapeutic regimen that inevitably becomes increasingly complex and burdensome. It is important that strategies are employed to minimize side-effects and that medications are monitored for both efficacy and safety. The crucial goal of PD treatments is the development of a neuroprotective treatment that slows disease progression [11]. Chinese medicine could represent a golden opportunity to discover novel and effective neuroprotective agents. The neuroprotection of StA, a dibenzocyclooctadiene lignan from the fruit of* Schisandra chinensis *(Turcz.) Baill, against two neurotoxins 6-OHDA and MPP^+^ was systematically investigated in our completed studies [9, 10]. The pharmacokinetic study and optimal formulation of StA are urgently needed. In recent years, pharmacokinetic studies on dibenzocyclooctadiene lignans, such as schizandrol A and schisandrin B, have been reported after administration of* schisandrae* extract or prescriptions [12]. The results have shown that these lignans are distributed and eliminated rapidly, and their bioavailabilities are relatively low; for instance, the absolute bioavailability of schizandrin was found to be 14.8% after oral administration in rat [12]. However, other lignan compounds, such as dibenzocyclooctadiene lignan (schisantherin A, Figure 2), have been rarely reported on pharmacokinetic study.

Poor bioavailability is one of major hurdles in drug development, particularly in the area of central nervous system (CNS) drugs [13]. Therefore, developing a novel approach by enhancing the drug delivery efficiency is urgently needed. In our previous studies, all our* in vitro* and* in vivo* data indicated StA's promising anti-PD activity, but further development of StA was required to address the problem of its poor bioavailability. The high dose of StA, at 300 mg/kg, exhibited significant and comparable effectiveness to 10 mg/kg of the anti-PD drug selegiline in our previous PD mice study [9, 10]. In terms of retaining MPTP-induced loss of TH-positive neurons of the SNpc in a PD mice study, the relatively lower potency of StA was probably because of the poor oral bioavailability of this compound. For the orally administered drug, there are two critical slower rate-determining steps in the bioavailability. First is the rate of dissolution and the rate of drug permeation through the membrane is another step. A recent review has summarized the formulation and delivery strategies of biopharmaceutical drugs [14]. Due to numerous advantages like transparency, ease of preparation, protection of labile drugs, increase of bioavailability, control of drug release, and increase of drug solubility can be associated with nanoemulsions [15]. Nanoemulsions have a higher surface area and free energy than macroemulsions and are stable against sedimentation, flocculation, coalescence, and creaming [16, 17]. Herein, an optimal nanoemulsion formulation which we developed significantly increases the bioavailability of StA to 47.3% (Table 2). The relative small particle size will be the reason to enhance the oral bioavailability; the excellent dispersion capabilities of nanoparticles in nanoemulsion were supposed to improve the solubilization of noncrystalline state of the drug in the matrix and the fast dissolution rate compared to pure drug suspension [18]. Moreover, reducing the size can modify surface properties of StA, the aqueous solubility and permeability through biological membrane [19], and the lymphatic transport through the transcellular pathway may also contribute to the increased bioavailability [20]. In addition, the long-chain triglycerides in the nanoemulsion components have been shown to promote the lymphatic absorption of the drugs from gastrointestinal tract which prevent the first-pass metabolism [21]. Recent literature data showed that nanoemulsions have been extensively used to increase the bioavailability of poorly soluble drugs. A number of drug candidates selected for full-scale development suffered from poor oral bioavailability mainly because of their limited solubilization. For example, resveratrol is a potent antioxidant that has shown good efficacy in the treatment of PD, but the main problem associated with the oral administration of this drug includes low bioavailability (less than 1%). Moreover, it is also unable to cross the BBB [22]. Recently, resveratrol was encapsulated in nanoemulsions to overcome the unfavorable pharmacokinetic properties [23–25]. The nanoemulsion formed from a self-nanoemulsifying drug delivery system resulted in an increase in the oral bioavailability of resveratrol by 430%, compared to resveratrol suspension [25]. These studies have demonstrated the ability of nanoemulsions to improve the poor water solubility and stability, the permeation, and the oral bioavailability of resveratrol. Another example is curcumin which has been shown to be effective against various diabetes related complications. However, a major limitation of curcumin is its low bioavailability. A nanotechnology based formulation called self-nanoemulsifying drug delivery system (SNEDDS) resulted in prolonged plasma exposure and bioavailability of curcumin [26]. The present study demonstrated that the use of nanoemulsions as vehicles for oral StA administration resulted in a significant enhancement of the StA oral bioavailability in rats in comparison with other two reagent preparation formulations (TPAP and DPS formulation). Based on the present results and the literatures above, nanoemulsion formulations appear to represent an attractive alternative that should be considered when there is a need to enhance the oral bioavailability of CNS drugs.

A previous study supported the notion that StA could pass through the blood-brain barrier (BBB) [27]. Furthermore, our pilot mice acute toxicity result demonstrated that 2 g/kg of StA, orally administered, did not produce any detectable toxicity (data not shown), indicating good tolerance of StA. Therefore, provided that the problem of poor absorption of StA in the gut is solved, the promising neuroprotective effects, kinetic drug metabolism, and safety profile of StA already suggest that StA fulfills the major requirements of a candidate for drug development. One further area of uncertainty concerns the contribution of metabolites of StA to the* in vivo* activity of StA, where a previous study reported that StA was subjected to liver metabolism* in vivo*; thus far, two StA metabolites, 7,8-dihydroxy-schisandrin, and an additional metabolite yet to be confirmed (with M.W. 432 g) have been identified in rat [28].

In conclusion, this study provides an example of advanced delivery system for poor soluble compounds like StA. This novel approach could overcome the shortcomings of conventional poor drug delivery of some CNS drugs. StA was prepared in nanoemulsion to satisfy well dispersion of the drug, to promote the dissolution and absorption, and finally to enhance the oral bioavailability. Our results confirmed that the nanoemulsion demonstrated the best performance compared with all other tested formulations. The pharmacokinetics of StA and a safe formulation of StA with high bioavailability had been determined. This data indicates that StA shall be a promising therapeutic agent for the prevention of PD by oral delivery.

## Figures and Tables

**Table 1:** Accuracy, extraction recovery, and calibration curve of StA in rat plasma (*n* = 5).

Biology samples	Concentration	Accuracy	Extraction recovery	Calibration curve
		(Mean ± SD, %)	(Mean ± SD, %)	
Plasma (*μ*g/mL)	25	101.9 ± 2.32	97.4 ± 1.51	*y* = 28.142*x* − 7.9608
	2.5	104.5 ± 4.41	93.7 ± 4.16	
	0.25	103.0 ± 1.79	88.3 ± 4.53	

**Table 2:** The main pharmacokinetic parameters of StA in the plasma of rats after intravenous or intragastric administration in four different ways (mean ± SD, *n* = 3).

Parameters	Unit	Formulation 1	Formulation 2	Formulation 3	
		Intragastric	Intravenous	Intravenous	Intragastric
AUC_(0−*t*)_	mg/L·h	18.38 ± 2.45	42.98 ± 4.59	67.67 ± 8.70	32.02 ± 12.51
MRT_(0−*t*)_	h	12.63 ± 1.24	8.58 ± 4.39	21.78 ± 2.24	11.46 ± 0.60
*T*_1/2*z*_	h	12.61 ± 2.82	12.19 ± 5.24	15.88 ± 1.82	16.54 ± 6.95
CLz	L/h/kg	14.19 ± 2.28	578.76 ± 112.33	359.37 ± 56.65	828.40 ± 238.27
Vz	L/kg	253.29 ± 68.98	9625.04 ± 2762.20	8135.47 ± 488.59	21330 ± 14355.2
*T*_max⁡_	h	4.167 ± 3.753	—	—	1.33 ± 0.58
*C*_max⁡_	mg/L	1.11 ± 0.42	—	—	2.73 ± 1.32
AUC_(0−*t*)_/dose	kg/L·h	0.0613	1.432	2.256	1.0675
Bioavailability	—	4.30%	—	47.30%	

## Abbreviations

MPP^+^: 1-Methyl-4-phenylpyridinium ion
MPTP: 1-Methyl-4-phenyl-1,2,3,6-tetrahydropyridine
6-OHDA: 6-Hydroxydopamine
Akt: Antiapoptotic kinase
CREB: Cyclic AMP response-element binding protein
DMSO: Dimethyl sulfoxide
PD: Parkinson's disease
PK: Pharmacokinetic
QC: Quality control
RSD: Relative standard deviation
SNpc: Substantia nigra pars compacta
StA: Schisantherin A
TH: Tyrosine hydroxylase.
